# Transsylvian opticocarotid resection of a stony craniopharyngioma with midbrain and ventricular extension

**DOI:** 10.3171/2026.7.FOCVID2673

**Published:** 2026-10-01

**Authors:** Omer Selcuk Sahin, Muhammed Yusuf Hamurcu, Samet Dinc

**Affiliations:** Department of Neurosurgery, Etlik City Hospital, Ankara, Turkey

**Keywords:** craniopharyngioma, calcified, pterional, transsylvian, opticocarotid corridor, zygomatic osteotomy

## Abstract

A 20-year-old male with a densely calcified craniopharyngioma involving the third ventricle and interpeduncular cistern underwent transsylvian resection with zygomatic osteotomy. After the lamina terminalis window proved inadequate, the approach was shifted to the opticocarotid corridor. The stony tumor required mechanical debulking with microscissors, Kerrison rongeurs, and pituitary curettes. A controlled "milking" maneuver delivered the soft ventricular component while preserving hypothalamic integrity. A small basilar artery remnant was intentionally left, achieving near-total resection. Postoperatively, visual fields remained intact; only transient diabetes insipidus occurred. This case illustrates intraoperative flexibility and mechanical debulking as a safe, effective strategy for heavily calcified craniopharyngiomas.

The video can be found here: https://stream.cadmore.media/r10.3171/2026.7.FOCVID2673

## Transcript

### 0:20 Patient Presentation and Clinical History.

A 20-year-old male presented with severe headache and increased intracranial pressure. After emergency EVD placement for obstructive hydrocephalus at an outside facility, he was transferred to our center for elective definitive resection following deliberate surgical planning.

### 0:37 Preoperative Imaging: CT.

CT reveals a densely calcified, "popcorn-like" suprasellar mass. The high Hounsfield units indicate a stony architecture, alerting us that ultrasonic aspiration would likely be insufficient and a mechanical debulking strategy must be planned.

### 0:52 Preoperative Imaging: MRI.

MRI shows superior invasion into the third ventricle and posterior interpeduncular extension with brainstem displacement. The third ventricular component required safe access without hypothalamic transgression; the interpeduncular extension demanded a low, wide-angle view of the basilar perforators. An endoscopic endonasal route could not reach the high third ventricular tumor.^1–3^ A standard pterional would lack inferior to superior angulation for the interpeduncular cistern.^4^ An interhemispheric translamina terminalis approach risked hypothalamic injury and poor access to the lateral calcification. Thus, a transsylvian approach with zygomatic osteotomy was chosen—providing simultaneous opticocarotid and interpeduncular access.^5,6^ The densely calcified core lay on the right, the softer cisternal portion on the left. A right-sided craniotomy gave direct access to the calcified core; after its removal, head rotation allowed safe reach to the contralateral component.

### 1:51 Opening and Approach.

The patient is positioned supine, head rotated 30° left and slightly extended to aid frontal retraction. A standard pterional incision is made, the temporalis reflected, and a one-piece zygomatic osteotomy performed;^5^ this low-angle, wide-base exposure minimizes brain retraction. After dural opening, sharp arachnoid dissection of the sylvian fissure mobilizes the frontal lobe, preserving bridging veins for early basal cistern visualization. The optic nerve and carotid are exposed by dividing the opticocarotid arachnoid bands.^7^ Using microscissors and blunt dissection, the tumor-carotid plane is developed, gently separating capsule from adventitia within the arachnoid plane. The third nerve is identified inferolaterally. This meticulous arachnoid dissection is the foundation for safe tumor access.

### 2:42 Corridor Selection and Intraoperative Strategy Shift.

Although the lamina terminalis is incised, this narrow and oblique window fails to provide surgical access to the tumor, which lies posterosuperiorly in the third ventricle, obscured by the chiasm and hypothalamus. Blind, angled dissection through this restricted opening would place the hypothalamus and perforators at unacceptable risk. Therefore, we immediately shift to the opticocarotid corridor for a direct, controlled trajectory. This intraoperative flexibility—abandoning an inadequate window for a safer alternative—is the central teaching point.

### 3:15 Management of Calcified Pathology.

Entering the opticocarotid space, the stony consistency is confirmed. Suction and bipolar are ineffective; ultrasonic aspiration fails even on high settings. We transition to mechanical fragmentation with microscissors, Kerrison rongeurs, and pituitary curettes. Force is controlled: scissors cut precisely to avoid torque, rongeurs close gently, curettes rotate to scoop without forward traction, minimizing transmitted force to the hypothalamus and perforators. The third cranial nerve is vigilantly protected. Gradual internal decompression allows the tumor capsule to collapse and mobilize.

### 3:50 Deep Cisternal Dissection.

After removing the calcified core, attention shifts to the softer posterior component adherent to Liliequist membrane. Sharp and blunt dissection separates the capsule, exposing the basilar artery and its perforators.^7^ Each perforator is preserved; the plane stays on the capsule surface—a high-stakes step protecting the posterior circulation. Opening the membrane reveals the basilar artery, superior cerebellar artery, and posterior cerebral artery confirming vascular integrity.

### 4:19 Third Ventricular Tumor Removal.

For residual soft tumor along the third ventricular floor, a controlled "milking" maneuver is used. The fragment is probed gently; only mobile, nonadherent portions are selected. Gentle external pressure delivers it through the opticocarotid corridor without axial hypothalamic traction. This is a standard microsurgical technique, not novel. It carries a recognized hypothalamic risk, which we mitigate by restricting the maneuver to clearly mobilized fragments and continuously monitoring the tumor-hypothalamic plane, preserving hypothalamic integrity.

### 4:53 Closure and Final Inspection.

Final inspection reveals a small remnant densely adherent to the basilar artery wall. Sharp dissection would risk vascular injury and brainstem ischemia; therefore, a planned near-total resection is accepted. Final survey confirms anatomical preservation of the optic chiasm, third nerve, and basilar trunk. The dura is closed with a multilayer duraplasty.

### 5:15 Postoperative Course and Outcomes.

Postoperatively, the patient developed expected diabetes insipidus, managed with desmopressin. Pituitary assessment confirmed isolated DI with intact anterior function. Neurological exam was normal; the patient was fully ambulatory. Formal visual field testing showed no pre- or postoperative deficits. MRI on day 1 confirmed near-total resection with only the small, intentional basilar remnant. Hydrocephalus resolved; the EVD was removed on day 4 without permanent CSF diversion. Surveillance MRI is planned every 3–6 months for 2 years. This case illustrates that for highly calcified craniopharyngiomas, an opticocarotid corridor with mechanical debulking is a safe, effective strategy.^8,9^

## Disclosures

The authors report no conflict of interest concerning the materials or methods used in this study or the findings specified in this publication.

## Author Contributions

Primary surgeon: Sahin. Assistant surgeon: Hamurcu, Dinc. Editing and drafting the video and abstract: all authors. Critically revising the work: Sahin, Hamurcu. Reviewed submitted version of the work: Sahin, Hamurcu. Approved the final version of the work on behalf of all authors: Sahin. Supervision: Sahin, Dinc.

## Supplemental Information

### Patient Informed Consent

The necessary patient informed consent was obtained in this study.
